# A porcine enterovirus G associated with enteric disease contains a novel papain-like cysteine protease

**DOI:** 10.1099/jgv.0.000799

**Published:** 2017-06-08

**Authors:** Todd P. Knutson, Binu T. Velayudhan, Douglas G. Marthaler

**Affiliations:** ^1^​ Veterinary Diagnostic Laboratory, Department of Veterinary Population Medicine, University of Minnesota, St Paul, MN 55108, USA; ^2^​ Texas A&M Veterinary Medical Diagnostic Laboratory, Amarillo, TX 79106, USA

**Keywords:** porcine, metagenomics, enterovirus G, *Torovirus*, papain-like cysteine protease, picornavirus

## Abstract

Identification of unknown pathogens in pigs displaying enteric illness is difficult due to the large diversity of bacterial and viral species found within faecal samples. Current methods often require bacterial or viral isolation, or testing only a limited number of known species using quantitative PCR analysis. Herein, faeces from two 25-day-old piglets with diarrhoea from Texas, USA, were analysed by metagenomic next-generation sequencing to rapidly identify possible pathogens. Our analysis included a bioinformatics pipeline of rapid short-read classification and *de novo* genome assembly which resulted in the identification of a porcine enterovirus G (EV-G), a complete genome with substantial nucleotide differences (>30 %) among current sequences, and a novel non-structural protein similar in sequence to the *Torovirus* papain-like cysteine protease (PL^pro^). This discovery led to the identification and circulation of an EV-G with a novel PL^pro^ in the USA that has not been previously reported.

## Abbreviations

EV-G, enterovirus G; FMDv, Foot-and-mouth disease virus; Lpro, picornavirus leader protease; NGS, next-generation sequencing; PLpro, papain-like cysteine protease; qPCR, quantitative PCR.

## Full-Text

Porcine viral outbreaks in the USA cause substantial economic losses to the swine industry. Rapid detection of common viral pathogens by quantitative PCR (qPCR) can be effective, yet these methods often fail to differentiate viral subtypes and cannot identify unknown viruses causing clinical disease [1]. Next-generation sequencing (NGS) of clinical samples has greatly enhanced our ability to identify or discover unknown pathogens in a variety of sample types [2, 3]. However, current methods are slow to identify unknown species in clinical samples, which can often require multiple laboratory and bioinformatics steps, such as: viral isolation, *de novo* assembly of all NGS reads and classification of contigs using blast. Herein, we have developed a metagenomics workflow to rapidly identify reads from NGS reads using Kraken, ultra-fast classification software comprising individual reads, and a custom database of known sequences [4]. This workflow identified and assembled an enterovirus G complete genome that contained a novel 669 nucleotide insertion from two diarrhoeic 25-day-old piglets at weaning from Texas, USA. This insertion encodes a protein similar to a *Torovirus* papain-like cysteine protease (PL^pro^), which resembles the picornavirus leader protease (L^pro^) [5].

The family *Picornaviridae* are single-stranded positive-sense RNA viruses that contain 31 genera, including *Aphthovirus* (including the species *Foot-and-mouth disease virus*, FMDv), *Senecavirus*, *Teschovirus*, *Sapelovirus* and *Enterovirus*. The genus *Enterovirus* comprises 12 species (enterovirus A–H and J, and rhinovirus A–C) [6]. The species *Enterovirus G* (EV-G) currently includes 16 serotypes/genotypes, where the two most common genotypes are EV-G1 (previously called porcine enterovirus 9) and EV-G2 (previously called porcine enterovirus 10) [6, 7]. Picornaviruses encode a single polyprotein that is proteolytically processed into three precursor protein products that are further processed into four structural proteins (VP1–4) and seven non-structural proteins (2A^pro^, 2B, 2C, 3A, 3B, 3C^pro^ and 3D-RdRp). Molecular subtyping based on VP1, VP4/2 or 3D sequence alignments have revealed a large diversity of EV-G genotypes (www.picornaviridae.com/enterovirus/ev-g/ev_g_seq.htm).

In 2016, the prevalence of EV-G infections in Vietnam was up to 90 % in piglets and 40 % in pigs over 1 year of age [8, 9], and the prevalence among Spanish swine herds at six farms ranged from 2 to 82 % [10]. A recent analysis found no difference in the prevalence of EV-G infection in pigs from Vietnam, with or without diarrhoea [8]. Indeed, EV-G has not been widely associated with pathogenic diseases in swine, except for single reports of skin lesions [11], flaccid paralysis [12] and the case (Porcine/USA/Texas1/2014, Porcine/USA/Texas2/2014) of diarrhoeic pigs described in this study. The substantially high prevalence and large number of current genotypes may suggest that EV-G-mediated pathogenesis requires confounding factors (e.g. co-infections), or that only certain genotypes trigger clinical manifestation. Finally, porcine EV-G circulation among the USA swine population has rarely been observed, except in one case from Minnesota [13].

A nursery in central Texas containing 21–55 day-old piglets was experiencing an outbreak of diarrhoea. Two faecal samples (Porcine/USA/Texas1/2014, Porcine/USA/Texas2/2014) were collected on 3 December 2014 and tested for common viral pathogens by qPCR or bacterial culturing, which did not reveal any potential sources of pathogens associated with the clinical outbreak. The samples were processed for NGS on the Illumina Miseq, using previously described methods [14, 15]. NGS generated an average of 680 000 reads per sample. The raw data were analysed using a custom pipeline of bioinformatics tools. Sequencing quality was assessed with FastQC v0.11.2 [16] and reads with highly repetitive sequences were removed using PRINSEQ-lite 0.20.4 [17]. Reads were trimmed (phred quality <20) and adapter/index sequences were removed using Trimmomatic v0.33 [18]. An average of 628 000 reads survived processing and each surviving read was taxonomically classified by Kraken software [4] using a custom Kraken database. The database was built using in-house and GenBank/RefSeq sequences (release 215, downloaded 23 November 2016). It included all GenBank viral and phage sequences, and the latest RefSeq archaea, fungi, protozoa, plasmid, plastid, mitochondrion and bacteria (complete, chromosome and scaffold) sequences. The RefSeq pig, human, corn and soybean (pig’s common food source) genomes were also included. All sequences were masked at low-complexity regions using dust v1.0.0 (NCBI blast +v2.2.28) [19], and the Kraken database was built with a *k*-mer size of 24 bp. This metagenomics bioinformatics pipeline identified the vast majority of eukaryotic and prokaryotic species in each sample, where only 8–10 % of reads in each sample were unidentified. These reads did not have any matches in the Kraken database and were subsequently investigated by *de novo* assembly and blast methods. The unknown contigs aligned to bacteria, bacteriophage, or pig sequences that were different from the genomes available from GenBank, and no other viral sequences were discovered. The Kraken-based classification revealed that a majority of reads in each sample were either host (2–37 %) or bacterial (49–58 %), and only 2.1–3.5 % of the reads were classified as viral. The majority of host or bacterial reads were derived from rRNA sequences, ranging from 6 to 62 %. The distribution of viral reads was explored for each sample and EV-G was the only virus detected, excluding bacteriophages. In each sample, 2743 (0.83 %) and 3856 (0.41 %) reads were classified as EV-G. Viral co-infection was nearly undetected in both samples, except that Porcine/USA/Texas1/2014 contained a small percentage of Sapporo virus (200 reads, 0.06 %).

**Fig. 1.**
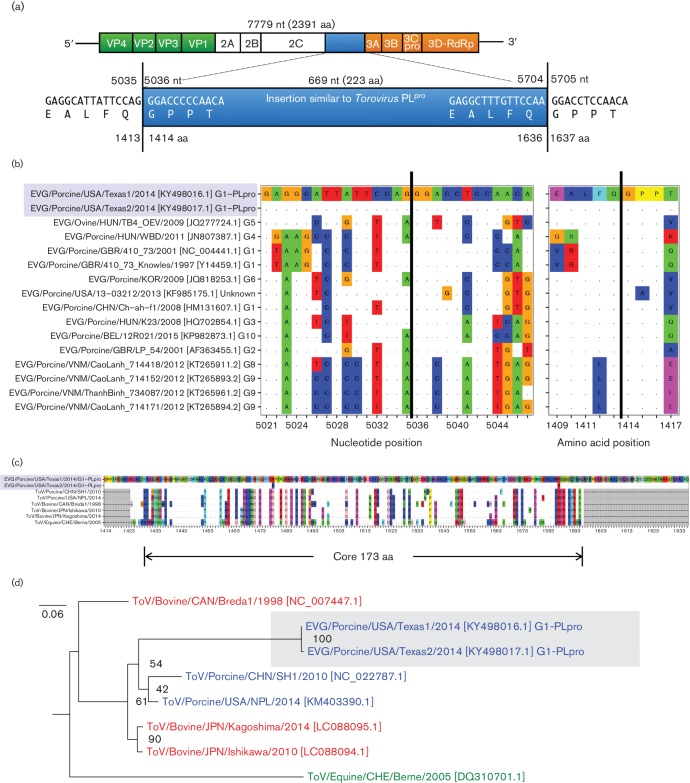
Genomic organization of the Texas EV-G stains and phylogenetic analysis of the insertion region. (a) The Texas EV-G viral ssRNA genomes contain a single ORF. These two strains contain a large 669 nt (233 aa) insertion (blue) with cleavage sites (EALFQ*GPPT) located at both ends of the insertion. The nucleotide (nt) and amino acid (aa) indices are shown, marking the boundaries of the insertion sequence. (b) The 3Cpro cleavage site between the 2C and 3A genes was analysed for nucleotide and amino acid conservation across all EV-G strains with complete genomes. The vertical black lines indicate where peptide cleavage occurs. The two Texas strains are highlighted in blue, and dots represent identical positions. Sequence positions correspond to the Texas1 strain. (c) Multiple sequence alignment of 223 aa Texas insertion and *Torovirus* strains revealing a core 173 aa region of considerable conservation, including multiple regions with 100 % aa identity. Sequence positions correspond to the Texas1 strain. GenBank accession numbers for each strain (top to bottom): KY498016.1, KY498017.1, NC_022787.1, KM403390.1, NC_007447.1, LC088094.1, LC088095.1 and DQ310701.1. (d) Mid-root phylogenetic tree of the Texas EV-G insertion nucleotide sequences aligned against six related *Torovirus* nucleotide sequences. Genomes are coloured according to their host species and labelled with strain names and Genbank accession numbers. Bootstrap support values are labelled as percentages.

*De novo* assembly of all the NGS reads per sample, which contained >92 % non-viral reads, is computationally intensive and time-consuming, and requires classification methods to identify the contigs of interest. Instead, the Kraken-based read classification method allowed rapid identification of the pathogenic virus, EV-G, and *de novo* assembly of only the EV-G-specific reads greatly increased genome assembly speed and quality. Using an assembly pipeline for analysis, the taxon-specific reads (EV-G) were isolated and *de novo* assembled with the Iterative Virus Assembler (iva) v1.0.6 [20], and the genomes were manually verified by remapping all sample reads using Bowtie2 v2.2.4 [21]. Each EV-G genome exhibited a mean coverage of 118×and 170× (min/max: 42×/231× and 58×/316×), respectively. Both complete EV-G genomes were 7999 nucleotides (nt) in length, encoding a single 2391 amino acid (aa) ORF (Fig. 1a). The assembly and verification pipeline generated accurate and complete EV-G genomes for both samples and the strain sequences were submitted to GenBank (EVG/Porcine/USA/Texas1/2014/G1-PLpro and EVG/Porcine/USA/Texas2/2014/G1-PLpro, accession numbers: KY498016 and KY498017, respectively).

Both EVG/Porcine/USA/Texas1/2014/G1-PLpro and EVG/Porcine/USA/Texas2/2014/G1-PLpro genomes were identical in length, but differed at five nucleotide positions, resulting in four non-synonymous amino acid changes (aa positions T5M, R1478H, K1587R and K2192E). The nucleotide and amino acid sequences of the Texas EV-G strains were searched against GenBank using blastn and blastp algorithms, which revealed a large insertion with a length of 669 nt (223 aa) found directly between the coding regions of 2C and 3A (from nt positions 5036 to 5704 and aa positions 1414 to 1636) (Fig. 1a). The alignment before and after the insertion sequence exhibited only 80 and 70 % nt identities (89 and 88 % aa identities) with the RefSeq EV-G strain UKG/410/73 (NC_004441). The Texas strains only exhibited 77 % nt identity with the only other USA EV-G strain USA/13-03212/2013 (KF985175.1), illustrating the substantial EV-G diversity between the current complete genomes, apart from the large insertion sequence discovered in the Texas strains.

The insertion sequence boundaries were verified computationally by remapping all sample reads to the full genome assembly and determining the overlapping read coverage across the boundaries. For each EV-G genome, the insertion boundaries had 88× and 132× read coverage, with individual read lengths up to 250 nt spanning the boundary, demonstrating that the insertion sequence was accurate and present in the original EV-G genome, and was not misassembled from an unrelated viral co-infection. The insertion sequence was also confirmed by PCR amplification across the entire region (from the 2C to 3A subunits), and verified by Sanger sequencing.

The unique 223 aa insertion sequence was queried against the GenBank protein database (blastp) and had 69 % aa identity (77 % query coverage) to the papain-like cysteine protease (PL^pro^) in ORF1a of *Torovirus*, strain PToV SH1 (YP_008798230.1). The insertion sequence belongs to the Peptidase_C28 (pfam05408) and Peptidase_C19 (cd02257) superfamilies. The Peptidase_C28 conserved protein domain family corresponds to the FMDv leader proteinase located at the N-terminus of FMDv polyprotein 1a, which cleaves the host translation initiation factor 4GI (eIF4G), preventing efficient 5′-cap-dependent translation and shifting host translation to the viral (cap-independent) translation [22]. The Peptidase_C19 domain family also has peptidase activity and is found in de-ubiquitination enzymes. Indeed, the PL^pro^ of other coronaviruses (e.g. SARS-CoV) are known to hydrolyse poly-ubiquitin chains, which may prevent degradation of viral proteins by the host proteasome [23]. However, the function of the PL^pro^ insertion in the Texas strains, and its contribution to viral replication, transmission and pathogenesis are still unknown.

During proteolytic processing of EV-G polyprotein, the 2C and 3A proteins are cleaved by the virus’s own 3C^pro^ protease at a well-defined cleavage sequence (EALFQ*GPPT) [24]. To investigate the insertion of the PL^pro^ in the Texas EV-G stains, a cleavage site analysis was performed by submitting the full-length ORF of the Texas EV-G strains to the NetPicoRNA 1.0 server [24], which predicted two highly probable cleavage sites (0.958/1.0 cleavage scores) flanking the insertion region (Fig. 1a). Thus, this analysis suggests that the PL^pro^ insertion sequence does not impact on the 2C and 3A proteins, is most likely proteolytically cleaved by 3C^pro^ from the precursor polyprotein and can function independently from the other prototypical EV-G proteins. Full-length ORFs from all current EV-G strains were analysed similarly (Fig. 1b). The nucleotide sequences in this cleavage site were substantially diverse, possibly to prevent spurious recombination events while total conservation sites were present at four aa positions (1411, 1413, 1414 and 1416), including the most critical residues, Q*G.

Next, the phylogenetic relationship between the PL^pro^ sequence from the Texas strains and the currently available *Torovirus* sequences in GenBank was investigated. A blastn search was run using the Texas strains as the query sequence, which identified only six *Torovirus*-related sequences with high significance (e-value <1e−16, query coverage >50 %). The blastn hits were translated and aligned with muscle v3.8.31 [25], revealing a core set of 173 aa conserved across all samples (Fig. 1c). This alignment showed multiple regions of perfect conservation, suggesting that the protease active sites are retained. Phylogenetic tree generation with nucleotide sequences was conducted with RAxML v8.2.8 [26] using maximum likelihood and a general time-reversible (GTR) substitution matrix with BFGS optimization and a gamma model with 1000 bootstraps (Fig. 1d). According to this tree, the PL^pro^ sequences from both Texas strains share a common ancestor with two porcine-derived *Torovirus* sequences, whereas the bovine and equine sequences were more distantly related. A closer phylogenetic relationship between the Texas EV-G insertion and the porcine *Torovirus* PL^pro^ sequences may suggest a common viral functionality of the PL^pro^-(like) protease in porcine. The phylogenetic tree distance (i.e. mean number of nucleotide substitutions per site) between the Texas insertion sequences (highlighted in blue) and the other two porcine strains (from China and the USA) was substantial, suggesting that if a recombination event occurred between the porcine (or other host-derived) *Torovirus* strains, these viruses have been evolving independently for some time after that event. Alternatively, the Texas EV-G strains may have acquired the PL^pro^ ‘insertion’ sequence through convergent evolutionary pressures that selected for a similar protease found among substantially different viruses within the families *Picornaviridae* and *Nidovirales* (e.g. FMDv and *Torovirus*).

**Fig. 2.**
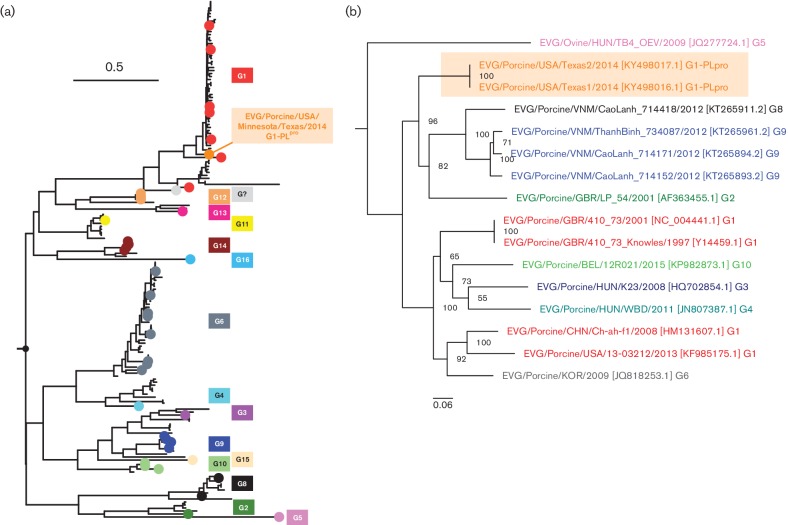
Phylogenetic analysis of all currently available EV-G VP1 sequences and complete genomes. (a) Phylogenetic analysis of all currently available VP1 EV-G nucleotide sequences. The tree was rooted at the midpoint and the coloured dots represent taxa that were previously classified using the genotype/serotype nomenclature (www.picornaviridae.com). The branch lengths between taxa are based on the mean number of nucleotide substitutions per site. The VP1 sequences from both Texas strains (orange) fall within the G1 (red) genotype clade. (b) Phylogenetic analysis of full-length EV-G nucleotide sequences. The tree was rooted at the midpoint and the genomes are coloured according to previously published genotypes (i.e. genotypes based on VP1 sequence trees). The strain names are followed by GenBank accession numbers and genotypes. The branch lengths between taxa are based on the mean number of nucleotide substitutions per site and bipartitions are labelled with bootstrapping support values (%). The two Texas strains are highlighted in orange.

The 14 currently available EV-G complete genomes from GenBank (with VP1-based genotypes: 1–6, 8–10) were identified from swine in the UK, Belgium, China, Hungary, Vietnam, South Korea and the USA [9, 13, 27–33]. Currently, EV-G genotypes are classified solely by the VP1. Phylogenetic analysis with all currently available EV-G VP1 sequences revealed that the Texas strains clustered in the middle of the large set of G1 genotypes (Fig. 2a), which suggests that the Texas strains may have evolved from a common G1 ancestor before acquiring the PL^pro^ insertion. Next, the phylogenetic relationships between all complete genomes were explored by aligning the nucleotide sequences and creating phylogenetic trees (as described above) (Fig. 2b). The two EV-G strains from Texas (highlighted in orange) share a common ancestor with the large clade containing EV-G genotypes G2, 8 and 9, but appear to be phylogenetically distant from the EV-G genotypes G1, 3, 4, 5, 6 and 10. The EV-G genotype 5 (derived from ovine) is most distantly related to the EV-G genomes from swine, suggesting that whole-genome reclassification may be needed after additional full-length genomes become available. Phylogenetic analysis, excluding the insertion sequence in the Texas strains, resulted in the same tree topology. Based on the complete-genome phylogenetic tree, the Texas strains appear to be distantly related to the other porcine EV-G sequences, whereas the VP1 tree suggests that the Texas strains are closely related to G1. Thus, this finding may indicate that the insertion sequence is more likely to have occurred through viral recombination (between a recent EV-G1 and a *Torovirus*), rather than it having been acquired through convergent evolution. However, additional complete genome sequences are needed to answer this question. Therefore, based on the relative differences in nucleotide identities between all current full-length EV-G genomes (>30 % different), and the discovery of a novel viral insertion, we propose listing the Texas strains as a EV-G genotype: G1-PL^pro^. The presence of only 14 full-length EV-G genomes makes the prevalence of the EV-G insertion sequence unknown, and it may also be present in other EV-G genotypes. A larger sampling of full-length EV-G genomes will reveal the global prevalence and provide evolutionary insights for this novel sequence.

In conclusion, we have developed a metagenomics sequencing and analysis approach for rapid identification of unknown pathogens in porcine samples. We identified and assembled an EV-G genome from the faeces of two 25-day-old piglets experiencing diarrhoea without any clear cause. To our knowledge, this is only the second time EV-G has been documented in the USA swine population and the Texas strains contain substantial nucleotide variation among related EV-G genotypes, yet the VP1 sequences are closely related to G1. In addition, the Texas strains identified herein include a unique viral protein not found in other EV-G viruses that may function similarly to the *Torovirus* papain-like cysteine protease, PL^pro^, with a genotype nomenclature of G1-PL^pro^.
